# Supplementary material for: Impact of Adverse Childhood Experiences (ACE) and Childhood Protective Factors (CPF) on physical and mental health of medical students of a public sector medical university
Source: PLOS Glob Public Health. 2025 Feb 24;5(2):e0004285. doi: 10.1371/journal.pgph.0004285 (PMC11849864; doi:10.1371/journal.pgph.0004285)
Supplement: Attachment — Submitted filename: Rebuttal letter.docx [file pgph.0004285.s002.docx]

To,

The Editor,

PLOS Global Public Health,

Subject: Rebuttal letter

Dear Sir/ Madam,

Thank you for giving this opportunity to revise the article. I am attaching my responses/ amendments below against each comment made:

1. Please provide additional details regarding participant consent. In the ethics statement in the Methods and online submission information, please ensure that you have specified (1) whether consent was informed and (2) what type you obtained (for instance, written or verbal, and if verbal, how it was documented and witnessed). If your study included minors, state whether you obtained consent from parents or guardians. If the need for consent was waived by the ethics committee, please include this information.

If you are reporting a retrospective study of medical records or archived samples, please ensure that you have discussed whether all data were fully anonymized before you accessed them and/or whether the IRB or ethics committee waived the requirement for informed consent. If patients provided informed written consent to have data from their medical records used in research, please include this information.

The following statement is entered

***Medical students over eighteen years of age who enrolled at King Edward Medical University were included in the study. Written consent was taken before inclusion in the study.***

2. We suggest you thoroughly copyedit your manuscript for language usage, spelling, and grammar. If you do not know anyone who can help you do this, you may wish to consider employing a professional scientific editing service.

Whilst you may use any professional scientific editing service of your choice, PLOS has partnered with both American Journal Experts (AJE) and Editage to provide discounted services to PLOS authors. Both organizations have experience helping authors meet PLOS guidelines and can provide language editing, translation, manuscript formatting, and figure formatting to ensure your manuscript meets our submission guidelines. To take advantage of our partnership with AJE, visit the AJE website (<http://learn.aje.com/plos/>) for a 15% discount off AJE services. To take advantage of our partnership with Editage, visit the Editage website ([www.editage.com](http://www.editage.com/)) and enter referral code PLOSEDIT for a 15% discount off Editage services. If the PLOS editorial team finds any language issues in text that either AJE or Editage has edited, the service provider will re-edit the text for free.

Upon resubmission, please provide the following:

- The name of the colleague or the details of the professional service that edited your manuscript

*Muhammad Shaheryar Khan*

- A copy of your manuscript showing your changes by either highlighting them or using track changes (uploaded as a *supporting information* file)

*Added*

- A clean copy of the edited manuscript (uploaded as the new *manuscript* file)

*Added*

3. In the online submission form, you indicated that "Will be available one request. Shall upload on github.".

All PLOS journals now require all data underlying the findings described in their manuscript to be freely available to other researchers, either 
1. In a public repository, 
2. Within the manuscript itself, or 
3. Uploaded as supplementary information.

This policy applies to all data except where public deposition would breach compliance with the protocol approved by your research ethics board. If your data cannot be made publicly available for ethical or legal reasons (e.g., public availability would compromise patient privacy), please explain your reasons by return email and your exemption request will be escalated to the editor for approval. Your exemption request will be handled independently and will not hold up the peer review process, but will need to be resolved should your manuscript be accepted for publication. One of the Editorial team will then be in touch if there are any issues.

*Uploaded on github (*[*https://github.com/Usmanali1994/ACEI*](https://github.com/Usmanali1994/ACEI)*)*

4. Please insert an Ethics Statement at the beginning of your Methods section, under a subheading 'Ethics Statement'. It must include:

1) The name(s) of the Institutional Review Board(s) or Ethics Committee(s)

2) The approval number(s), or a statement that approval was granted by the named board(s)

3) (for human participants/donors) - A statement that formal consent was obtained (must state whether verbal/written) OR the reason consent was not obtained (e.g., anonymity). NOTE: If child participants, the statement must declare that formal consent was obtained from the parent/guardian.

The following statement is added

*Ethics Statement: Ethical approval was taken from the Institutional Review Board of King Edward Medical University, Lahore, Pakistan. (Letter no. 318/ RC/ KEMU dated 18 May 2024).*

5. Please provide an Author Summary. This should appear in your manuscript between the Abstract (if applicable) and the Introduction, and should be 150–200 words long. The aim should be to make your findings accessible to a wide audience that includes both scientists and non-scientists. Sample summaries can be found on our website under Submission Guidelines:

<https://journals.plos.org/globalpublichealth/s/submission-guidelines#loc-parts-of-a-submission>

*The current study was carried out to evaluated the impact of childhood adverse experiences and childhood protective factors on physical and mental health among medical students. We conducted a cross-sectional study at the King Edward Medical University, Lahore, Pakistan. Participants were interviewed using a questionnaire comprising of Adverse childhood experiences (ACE), childhood protective factors (CPF), and self reported physical and mental health. A total of three hundred and sixty eight participants, which 125 (33.96%) were male and 243 (66.93%) were females. About 89.5% medical students had three or more adverse childhood experiences. In terms of sexual abuse18.4% of male medical students reported facing sexual abuse compared with 11.1 % of female students. Higher Childhood Protective Factor (CPF) score predicted physical health at good, and excellent level. Higher ACE score was a significant predictor of mental health at fair level, good and excellent levels. CPF score was also a significant predictor of mental health at good and excellent levels.*

6. We have noticed that you have cited Supporting Information files in your manuscript's Data availability on page 15. However, there are no corresponding files uploaded to the submission. Please upload them as separate files with the item type 'Supporting Information'. 

Please review your reference list to ensure that it is complete and correct. If you have cited papers that have been retracted, please include the rationale for doing so in the manuscript text, or remove these references and replace them with relevant current references. Any changes to the reference list should be mentioned in the rebuttal letter that accompanies your revised manuscript. If you need to cite a retracted article, indicate the article’s retracted status in the References list and also include a citation and full reference for the retraction notice.

Additional Editor Comments (if provided):

This is an important and well-written study.

My concern is that it is limited to one private medical college therefore the results cannot be generalized. This should be mentioned in the Limitations section. In addition, there should be some mention of the applicability of these results from a public health perspective.

The following statement is added:

*Our study was conducted in one medical university students; thus, generalization of results could not be made.……. Given the stigma associated with childhood adversities university may include awareness and de-stigmatization campaigns so that students can open up their experiences and can seek mental health services.*

Thank You

Dr. Usman Ali,

Corresponding author	Dated: January 8, 2024.
